# Comprehensive genomic analyses revealed the adaptation strategies of *Exiguobacterium* and its phage genomic diversity

**DOI:** 10.3389/fmicb.2026.1841508

**Published:** 2026-05-21

**Authors:** Yiping Tian, Leyi Zou, Yu Ji

**Affiliations:** 1 Department of Geography, Xinzhou Normal University, Xinzhou, China; 2 First Clinical Medical College, Gansu University of Chinese Medicine, Lanzhou, China; 3 Lab for Microbial Resources, School of Ecology and Environment, Inner Mongolia University, Hohhot, China

**Keywords:** comparative genomic analysis, environmental adaptability, *Exiguobacterium*, genetic diversity, horizontal gene transfer (HGT)

## Abstract

*Exiguobacterium* exhibits high species diversity and complex evolutionary patterns, with members widely distributed across diverse habitats. To elucidate the mechanisms enabling its high adaptability to various environments, 187 genomes of *Exiguobacterium* strains were analyzed using the phylogenomic and comparative genomics methods. Our analysis revealed that nearly all *Exiguobacterium* strains harbor genes encoding the utilization of diverse complex polysaccharides and proteinaceous, as well as intact glycolysis and tricarboxylic acid cycle pathways. These abilities suggest that the strains of this genus can easily obtain carbon and nitrogen from the environment. Furthermore, *Exiguobacterium* strains encode heat- and cold-shock proteins for temperature adaptation, accumulate potassium and compatible solutes such as mannitol, betaine, glutamate, and proline for osmotic balance, and synthesize antioxidant enzymes including superoxide dismutase, catalase, peroxidase, disulfide isomerase, and methionine sulfoxide reductase to mitigate oxidative stress. Each *Exiguobacterium* strain also contains many genes for resistance to antibiotics and heavy metals, many of which are identified within genomic islands, indicating that horizontal gene transfer has substantially contributed to the rapid acquisition and spread of these adaptive traits. In addition, the presence of diverse phages further enhances genomic variability, and the identification of three auxiliary metabolic genes indicates a potential role for these phages in modulating specific host metabolic processes during infection. This study enhances our understanding of the adaptive mechanisms and key genomic traits of *Exiguobacterium* that enable its cosmopolitan distribution.

## Introduction

*Exiguobacterium* is a Gram-positive, facultatively anaerobic genus that has been widely detected and isolated from diverse habitats, including soil (Singh et al., 2013), marine sediment (Dastager et al., 2015), marine algae (Kasana and Pandey, 2018), freshwater (Raichand et al., 2012), plant rhizospheres (Vishnivetskaya et al., 2009), industrial effluents (Alam and Malik, 2008), and extreme environments such as salt lakes (Jiang et al., 2013), glaciers, permafrost (Rodrigues et al., 2008), and hydrothermal vents (Crapart et al., 2007). Members of this genus exhibit remarkable physiological versatility, with growth temperatures ranging from −12°C to 55°C, pH tolerance from 5 to 12, and salinity tolerance from 0% to 19% (NaCl, m/v; Shen et al., 2024; Vishnivetskaya et al., 2009). The extensive ecological distribution and genetic diversity of *Exiguobacterium* make it an excellent model for investigating the genomic basis of environmental adaptation (Kasana and Pandey, 2018; Zhang et al., 2021).

Beyond its ecological significance, the genus also exhibits considerable applied potential. Several species have been reported to produce industrially relevant enzymes, particularly hydrolytic enzymes such as proteases, lipases, cellulases, amylases, and pullulanases, which are widely utilized across industries including detergents, food, feed, dairy, paper, textile, leather, and pharmaceuticals (Kasana and Pandey, 2018). In addition, multiple *Exiguobacterium* species have demonstrated capabilities in biodegradation and bioremediation. For example, *Exiguobacterium indicum* and *Exiguobacterium* sp. GS1 have been reported to tolerate and remove heavy metals such as chromium and arsenic from contaminated environments (Okeke, 2008; Rodríguez-Castrejón et al., 2022), while *Exiguobacterium aurantiacum* has shown potential in the degradation of organic pollutants (Jeswani and Mukherji, 2012). Six strains of *Exiguobacterium aurantiacum* and *Exiguobacterium* sp. AT1b/GX59, have also been reported as opportunistic bacteria in clinical settings, suggesting potential implications for human health and antimicrobial resistance monitoring (Chen et al., 2017; Pitt et al., 2007). Importantly, understanding the genomic basis of these functional traits is essential for unlocking the full applied potential of *Exiguobacterium*. Genome-resolved analyses can reveal key genes and pathways underlying stress tolerance, thereby clarifying how these bacteria adapt to extreme environments and providing a genetic basis for potential applications. Harnessing these adaptive traits, together with identified genes for pollutant degradation and enzyme production, can accelerate the discovery of novel biocatalysts, strengthen bioremediation strategies, and enable the development of microbial strains with enhanced performance under harsh industrial settings.

With the rapid increase in available genome sequences, comparative genomic studies have begun to elucidate the genetic basis underlying the environmental adaptability of *Exiguobacterium*. Previous studies have identified diverse carbohydrate-active enzymes (CAZymes), extracellular peptidases, and genes involved in stress response systems related to temperature, salinity, and pH adaptation (Vishnivetskaya et al., 2009; Zhang et al., 2021), providing important insights into the metabolic flexibility and ecological success of this genus. However, most existing studies have focused on a limited number of strains or specific functional traits, lacking a comprehensive and integrative evaluation. In particular, key adaptive traits such as oxidative stress response, heavy metal tolerance, and antibiotic resistance remain insufficiently explored at the genomic level, limiting a systematic understanding of the mechanisms underlying the broad ecological distribution and environmental resilience of *Exiguobacterium*.

In addition to chromosomal adaptive traits, mobile genetic elements (MGEs) are recognized as important drivers of bacterial genome evolution and functional diversification (Tokuda and Shintani, 2024). Among them, bacteriophages play a central role in mediating horizontal gene transfer (HGT), shaping genome plasticity, and influencing host ecological fitness (Brussow et al., 2004). During infection, phages can reprogram host metabolism through the expression of viral-encoded auxiliary metabolic genes (AMGs), while phage-mediated HGT further modulates host fitness, thereby promoting microbial evolution and diversification (Rohwer et al., 2009). Once integrated into bacterial genomes, phages may become stable genomic components that contribute to genome expansion, structural variation, and long-term genetic diversification (Canchaya et al., 2003). Despite the broad ecological distribution of *Exiguobacterium*, the diversity, genomic characteristics, and potential functional roles of prophages within this genus remain poorly understood. A systematic assessment of prophage diversity and their potential contributions is therefore crucial for understanding the genomic dynamics and adaptive capacity of *Exiguobacterium*.

To address the remaining gaps in understanding the genomic basis of environmental adaptation in *Exiguobacterium*, we performed a comprehensive comparative genomic analysis of 187 *Exiguobacterium* genomes. We aimed to: (i) elucidate the fundamental metabolic capabilities of the genus; (ii) systematically characterize genes associated with adaptation to extreme environmental conditions; and (iii) explore the diversity of *Exiguobacterium* phages and their interactions with host. By integrating large-scale comparative genomics with prophage analysis, this study advances our understanding of the genetic basis of environmental adaptation in *Exiguobacterium* and provides valuable genomic resources for future applications in biotechnology, environmental remediation, and microbial engineering.

## Methods

### Genome sources and analysis of the *Exiguobacterium* strains

By July 2024, there were 327 *Exiguobacterium* strains (including culture isolates and metagenome-assembled genomes) with published genome sequences in the GenBank. To ensure the accuracy of the analysis, high-quality (completeness ≥95% and contamination ≤ 5%) genomes evaluated by CheckM v1.1.3 (Parks et al., 2015) were dereplicated at 99.99% ANI using dRep v3.4.0 (Olm et al., 2017) (parameters: –S_algorithm ANImf -comp 95 -con 5 -sa 0.9999). Only 187 strain-level genomes of *Exiguobacterium* were selected for further phylogenomic and genomic analyses (Supplementary Table S1). Among these genomic sequences analyzed, 53 were assigned to 17 different *Exiguobacterium* species and 134 were not accurately identified to species. To evaluate the accurate taxonomic position of *Exiguobacterium*, ANI and dDDH values between these strains were calculated using fastANI v1.33 (Jain et al., 2018) and the Genome to Genome Distance Calculator (GGDC 3.0; http://ggdc.dsmz.de/ggdc.php; Auch et al., 2010), respectively.

The pan and core genomes of 187 *Exiguobacterium* strains were analyzed using the Bacterial Pan-Genome Analysis Tool (BPGA) pipeline v1.3 with default parameters (Chaudhari et al., 2016). In a pan-genome analysis, the number of accumulated genes that are related to the number of genomes can be predicted by Heaps' law as *n* = *a* × *x*^*b*^, when 0 < *b* < 1 indicates that the pan-genome is open, while *b* < 0 is closed (Tettelin et al., 2008).

### Metabolism analysis

The primary metabolic profiles and secondary metabolite biosynthesis gene clusters (smBGCs) were analyzed using KAAS (KEGG Automatic Annotation Server) and antiSMASH version 7.0 (https://antismash.secondarymetabolites.org/; Blin et al., 2023), respectively. Draft or contig-level genomes may yield incomplete primary metabolic pathway annotations because key genes can be missing, and genes belonging to the same smBGC can be fragmented across multiple contigs, leading to an overestimation of the number of predicted clusters. Therefore, only 18 complete genomes of *Exiguobacterium* were used to ensure the accuracy of both primary metabolic pathway reconstruction and smBGC prediction. A metabolic pathway was considered complete if all required KO entries for each enzymatic step were present in the genome, and considered incomplete or lacking if one or more essential enzymatic steps (i.e., required KOs) were absent. The carbohydrate active enzymes (CAZymes) of 187 *Exiguobacterium* genomes were identified using the HMMER method of the dbCAN3 online server (https://bcb.unl.edu/dbCAN2) with the parameters *e*-value ≤ 1*e*−15 and coverage >0.35 (Zheng et al., 2023). The proteases were classified with Diamond BLASTP using the MEROPS database (Rawlings et al., 2018), with a cutoff *e*-value of ≤ 1*e*−5.

### Search for functional genes and genomic islands in the genomes

The genes related to abiotic stresses were firstly searched in 18 complete genomes, and the sequences obtained were compared with the reference sequences in GenBank and UniProt to determine the correctness of the sequences. Subsequently, the obtained sequences were used as the reference sequences to search for their counterparts in the remaining genomes using BLASTP (http://blast.ncbi.nlm.nih.gov/Blast.cgi). Only sequences that had >40% identity and >50% coverage with the reference sequences indicated that the genome contained the gene (Zhao et al., 2023).

The antibiotic resistance genes were identified using the resistance gene identifier (RGI) of the CARD (https://card.mcmaster.ca/; Alcock et al., 2023), and the sequences with Perfect, Strict, and Loose hits identities >50% were selected for further analysis. The genomic islands (GIs) of the genomes were predicted using the IslandViewer 4 online server (http://www.pathogenomics.sfu.ca/islandviewer/) with the IslandPick, SIGI-HMM, IslandPath-DIMOB, and Islander methods (Bertelli et al., 2017).

### Identification and taxonomic assignment of phages associated with *Exiguobacterium*

Prophage-like sequences in *Exiguobacterium* genomes were predicted using a combination of four virus-finding tools, including PHASTEST online server (https://phastest.ca; Wishart et al., 2023), geNomad v1.8.0 (parameters: end-to-end) (Camargo et al., 2024), VirSorter2 v2.2.4 (parameters: –min-length 5,000 –min-score 0.8; Guo et al., 2021) and VIBRANT v1.2.1 (parameters: default; Kieft et al., 2020). Putative viral contigs that were identified as higher confidence predictions (i.e., “intact” or “questionable” in PHASTEST, “score > 80” in geNomad and Virsorter2, and “complete,” “high-,” or “medium-quality” in VIBRANT) by at least two methods were retained for further analysis. In addition, uncultivated viral genomes (UViGs) potentially infecting *Exiguobacterium* strains were retrieved from high-confidence viral genomes (~5.5 million sequences) in the IMG/VR v.4 dataset (Camargo et al., 2023). *Exiguobacterium*-associated UViGs were further validated using iPHoP v1.3.3 with the “Aug_2023_pub” host database and default “predict” parameters (Roux et al., 2023).

The prophage and UViGs associated with *Exiguobacterium* obtained were pooled together and only viral Contigs ≥5 kb was retained. These Contigs were then clustered at 95% ANI and 80% coverage using the CheckV script (https://bitbucket.org/berkeleylab/checkv/src/master/) to generate non-redundant viral operational taxonomic units (vOTUs). The quality of the vOTUs was estimated using CheckV v1.0.3 (Nayfach et al., 2021), which classified sequences into five quality tiers: complete (100% completeness), high quality (completeness ≥90%), medium quality (completeness 50-90%), low quality (completeness 0%−50%), and not-determined quality (no completeness estimate available).

The taxonomy of UViGs was retrieved from the IMG/VR v.4 database and prophages clustered with them in vOTU were assigned to the same taxon. For the remaining phages, two classification methods were used: (i) Marker protein profile-based taxonomic assignment using geNomad v1.8.0 with the default parameters. (ii) The family-level taxonomic classification of vOTUs was conducted using PhaGCN v2.3 (Jiang et al., 2023) based on the latest International Committee on Classification of Viruses (ICTV, 2024) taxonomy profile. Obtained virus families classified as “Family_like” were considered unclassified.

### Phylogenetic analysis

The core genome of *Exiguobacterium* was constructed by applying a cut-off value of 50% sequence identity using USEARCH. After the resulting core genes were concatenated and aligned using MUSCLE5 (Edgar, 2022), a phylogenetic tree was constructed based on the concatenated core genes using MEGA v.6.0 with the maximum likelihood (ML) method (Tamura et al., 2013). The tree topology was evaluated using the bootstrap analysis based on 1,000 resampling replicates. Sequences of previously reported MCPs were used as references in the phylogenetic analysis of DJR MCPs (Yutin et al., 2018). For AMGs, homologous sequences were retrieved from the NCBI nr database using BLASTP, and the top 20 hits with a bit score threshold of 50 were retained as reference sequences for AMGs. The amino acid sequences were aligned using MUSCLE5, and manually checked and trimmed. The phylogenetic trees were constructed using the methods described above and visualized using the iTOL online server (https://itol.embl.de/; Letunic and Bork, 2021).

*Exiguobacterium* phages were classified with the virus-host DB (RefSeq release 220) based on genome-wide similarity using ViPtree v4.0 (https://www.genome.jp/viptree/). The relevant phages with genome similarity scores (SG) > 0.02 were selected to generate proteomic tree based on genome distance matrix using BIONJ algorithm (Nishimura et al., 2017).

### Identification of auxiliary metabolic genes (AMGs)

Viral AMGs were identified and annotated using DRAM-v v1.4.6 (Shaffer et al., 2020) and VIBRANT v1.2.1 (Kieft et al., 2020). (i) DRAM-v pipeline. The AMGs were searched for in vOTUs using DRAM-v with the KEGG, UniRef-90, Pfam, dbCAN, VOGDB, and the MEROPS peptidase databases. Only putative AMGs with an auxiliary score < 4 and a gene ID or gene description were retained. (ii) VIBRANT pipeline. Prediction of AMGs in vOTU using VIBRANT with default parameters. To improve confidence in AMG identification, AMGs predicted by the two pipelines were merged and manually curated to remove genes related to nucleotide metabolism, organic nitrogen, transcriptional/translational regulators, ribosomal proteins, structural proteins, viral invasion (i.e., glycoside hydrolases and peptidases involved in cell wall lysis), and viral component modifications (i.e., glycosyl transferases, adenylyltransferases, and methyltransferases; Luo et al., 2022). In addition, putative AMGs that were inconsistently annotated in different databases were manually corrected to “putative protein” to improve annotation accuracy. The genome maps of vOTUs encoding DJR MCPs and AMGs were drawn using gggene (https://github.com/wilkox/gggenes).

## Results

### General features and phylogenetic analysis of the *Exiguobacterium* strains

**Figure 1:**
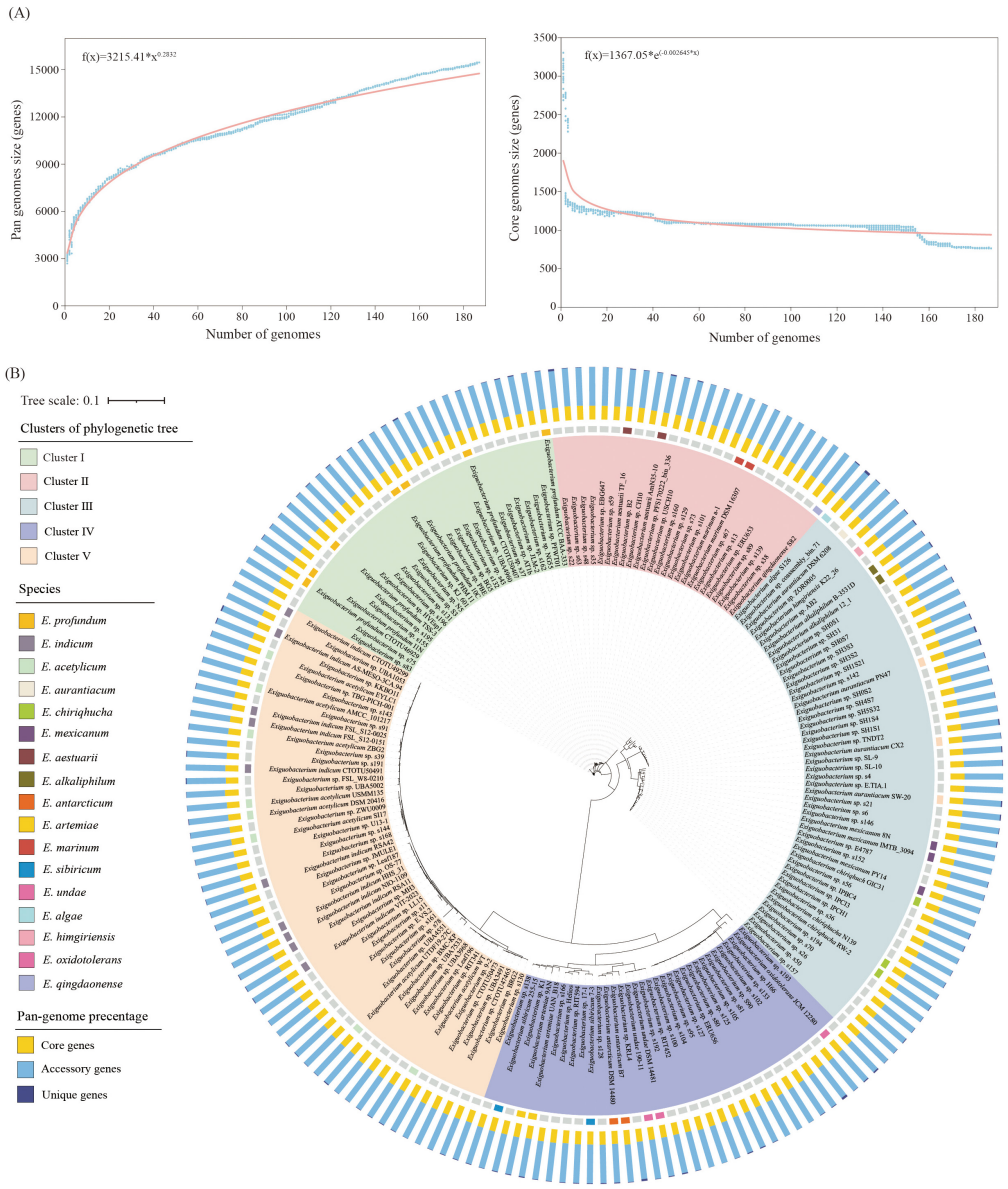
**(A)** Pan-genome and core genome plots of the 187 strains of *Exiguobacterium*. The plot shows the equations fitting the total and core gene families, as well as how the number of gene families increase and decline in the pan and core genome with each consecutive addition of a *Exiguobacterium* genome. **(B)** Phylogenetic tree constructed based on core genomes. The three colors in the bar graph represent the percentage of core, accessory, and unique genes in the pan-genome.

The genome size of the 187 strains of *Exiguobacterium* ranged from 2.6 to 3.4 Mb with a G+C content of 45.9%−53.1%. The number of coding sequences (CDS) ranged from 2,613 to 3,440. The pan-genome of 187 *Exiguobacterium* strains contained 15,471 gene families, with 763, 9,827 and 4,881 core, accessory and unique genes, respectively. According to Heaps' law, the pan-genome of *Exiguobacterium* was open and increasing (*b* = 0.29; Figure 1A). In addition, 187 strains were distributed in 17 different *Exiguobacterium* species (Figure 1B), 11 of which could be found in diverse habitats (Supplementary Table S1).

Analysis of the phylogenetic tree based on core genes suggested that 187 strains of *Exiguobacterium* can be assigned to five clusters. Clusters I, II, III, IV, and V are composed of one, three, six, five, and two species, respectively (Figure 1B). The ANI values between all the strains tested were higher than 76%, indicating that these strains of *Exiguobacterium* belonged to a single genus (Supplementary Figure S1). The topology of the tree constructed using the ANI values is almost similar to that based on the core genome (Figure 1B; Supplementary Figure S1).

Based on the species classification thresholds (ANI-values > 95% and dDDH-values > 70%; Chun et al., 2018), 44 of the 134 unclassified genomes could be assigned to 10 species (Supplementary Table S2). In addition, comparison with type strains revealed that several genomes were likely misclassified, as their ANI and dDDH values were consistently below the species delineation thresholds. For example, strains AmN35–10 and 7–3, previously assigned to *E. aestuarii* and *E. sibiricum*, respectively, showed ANI values of 90.9 and 90.4% and dDDH values of 41.8 and 38.9% relative to their corresponding type strains, indicating potential misclassification at the species level. Similarly, several genomes assigned to *E. aurantiacum, E. indicum*, and *E. acetylicum* also exhibited below-threshold genomic similarity to their type strains, further suggesting misidentifications in public databases (Supplementary Table S2).

### Metabolic analyses of *Exiguobacterium* strains

**Figure 2:**
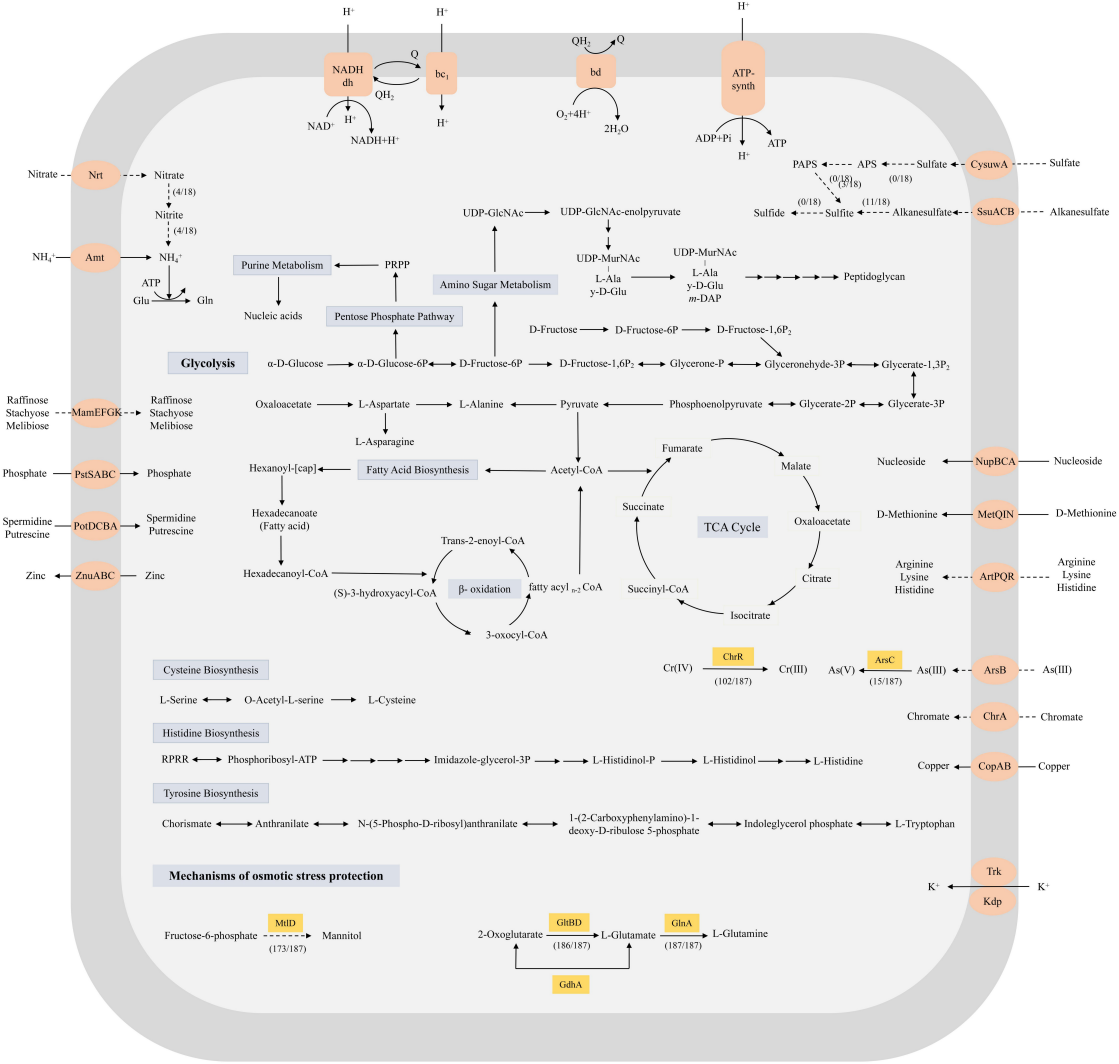
Prediction of the central metabolic potential of 18 complete genomes of *Exiguobacterium* strains, and the mechanism of osmotic stress adaptation of the 187 strains. Solid lines, pathways common to all strains; dashed lines, partial presence.

To characterize the core metabolic capabilities of *Exiguobacterium* and explore potential metabolic limitations related to environmental adaptation, all 18 complete genomes were subjected to KEGG analysis. The results revealed that all 18 strains harbored intact glycolysis, tricarboxylic acid cycle (TCA cycle), pentose phosphate metabolism, fatty acid and peptidoglycan biosynthesis pathways, but lacked complete sulfur and nitrogen metabolism pathways (Figure 2; Supplementary Table S3). Specifically, enzymes involved in dissimilation sulfate reduction and sulfur oxidization (Sox) system were not detected in all 18 strains. The *cysH* gene, which converts 3′-Phosphoadenylyl sulfate (PAPS) to sulfite, was found only in *Exiguobacterium* sp. AT1b, *Exiguobacterium* sp. PFWT01 and *E. marinum* a-1. For nitrogen metabolism, nitrate reductase (NasA), which converts nitrate to nitrite, was only detected in *Exiguobacterium* sp. AT1b, *Exiguobacterium* sp. PBE, *Exiguobacterium* sp. PFWT01 and *E. aurantiacum* CX2, while enzymes related to the conversion of nitrite to ammonia, nitrification, denitrification, nitrogen fixation, and anammox were absent in all strains. Additionally, all 18 strains of *Exiguobacterium* were able to synthesize nine types of amino acids, including alanine, aspartate, asparagine, glutamate, glutamine, cysteine, arginine, histidine, and tryptophan.

The phosphotransferase system (PTS) is the key signal transduction pathway involved in the optimal utilization of carbohydrates by bacteria in complex environments and consists of two cytoplasmic energy-coupled proteins (Enzyme I and HPr) and a series of carbohydrate-specific Enzymes II (EII; Kotrba et al., 2001). The genomic analysis revealed that all strains harbored the *ptsI* and *ptsH* genes that encode EI and HPr, respectively, as well as seven genes belonging to the carbohydrate-specific EII, which were involved in the translocation and phosphorylation of carbon sources, including glucose (*ptsG* and *crr*), N-acetyl-D-glucosamine (*nagE*), sucrose (*scrA*), β-glucoside (*bglF*), and trehalose (*treB* and *treP/crr*; Supplementary Table S3). In addition, all 18 *Exiguobacterium* strains harbored the *flg* operon (flagellar motif), the *fli* cluster, and *flhAB* encoding flagellum structural proteins, as well as the *motAB, cheACDRVWY*, and *mcp* genes encoding the chemotaxis proteins, which enabled the rapid cellular response to environmental changes. The two-component system genes related to phosphate limitation (*phoRPA*), oxygen limitation (*resDE* and *catA*), salt stress (*degSU*), and cell wall-active antibiotics/cationic antimicrbial peptidewere (*liaFRS*) were also found in the core genome, which enabled the *Exiguobacterium* strains to more efficiently adapt to complex and variable environments.

Secondary metabolites are organic molecules that are not required for basic survival, but they can enhance the environmental adaptability of the strain (O'Connor, 2015). A total of 36 smBGCs of two major types were predicted in the 18 *Exiguobacterium* genomes, and each strain contained one to three smBGCs (Supplementary Table S4). Among them, terpene-related smBGCs were found in all 18 *Exiguobacterium* strains, demonstrating their potential for a variety of bioactivities, including antioxidant, antimicrobial, antifungal, antiparasitic, and antiviral (Paduch et al., 2007). The clusters responsible for the biosynthesis of siderophores, which could enhance microbial iron uptake under iron-limiting conditions (Saha et al., 2016), were found in seven strains. Notably, 18 terpene BGCs were not similar to any known gene clusters, suggesting that they may be the new types of terpenes.

### Diversity of CAZymes and peptidases in *Exiguobacterium* strains

Carbohydrates are the main carbon source for microorganisms and their metabolism is catalyzed by carbohydrate active enzymes (CAZymes). A total of 73 different CAZyme families belonging to six CAZyme superfamilies were identified from 187 *Exiguobacterium* genomes, including 43 glycoside hydrolases (GHs), 12 glycosyltransferases (GTs), 7 carbohydrate esterases (CEs), 5 carbohydrate-binding modules (CBMs), 4 auxiliary activities (AAs), and 2 polysaccharide lyases (PLs; Supplementary Figure S2). Each genomic sequence encoded between 61 and 92 CAZymes (average 76).

The analysis of CAZymes involved in the degradation of complex polysaccharides revealed that categories related to the degradation of hemicellulose, cellulose, starch, and chitin were enriched in the genome of *Exiguobacterium*. Specifically, all strains produced GH31-family enzymes that degraded hemicellulose, while only some of the 187 strains harbored GH16- (135 strains), GH42- (130 strains), and GH43-family (54 strains) enzymes involved in the degradation of hemicellulose. The most numerous families involved in cellulose degradation are GH1 (average of more than four genes per genome) and GH3 (average of more than two genes per genome). The GH13 (187 strains) and GH57 families (186 strains) represent the main starch-degrading enzyme families. Among the GH13 family members, GH13_5 (α-amylase), GH13_14 (pullulanase), GH13_29 (α-glucosidase), and GH13_31 (α-glucosidase) were present in all strains. The GH23 (chitinase), CE4 (deacetylase), and CBM50 (chitin binding) families, which are responsible for the degradation of chitin, were detected in 186, 187, and 184 strains, respectively. In addition, the GH65 (trehalose phosphorylase) family related to trehalose utilization was found in 186 strains of *Exiguobacterium*.

Proteinaceous compounds are abundant forms of organic nitrogen in the environment, and extracellular microbial peptidases play a crucial role in degrading them (Nguyen et al., 2019). In total, 14,065 putatively secreted peptidases were identified and assigned to 45 families (59 subfamilies); of them, 17, 14, seven, three, one, one, one, and one belonged to the metallo, serine, cysteine, aspartic, glutamic, asparagine, threonine, and unknown catalytic type peptidase families, respectively (Supplementary Figure S3). Each genomic sequence encoded between 67 and 84 peptidases (average 75). Among these secreted peptidases, the C26 (gamma-glutamyl hydrolase), S33 (prolyl aminopeptidase), S09 (prolyl oligopeptidase), and M38 (isoaspartyl dipeptidase) represented the top four most abundant peptidases (Supplementary Figure S3). The presence of numerous CAZymes and peptidases revealed that *Exiguobacterium* strains were able to metabolize and utilize a wide range of polysaccharides and proteins from the environment.

### The ability to adapt to abiotic stresses

**Figure 3:**
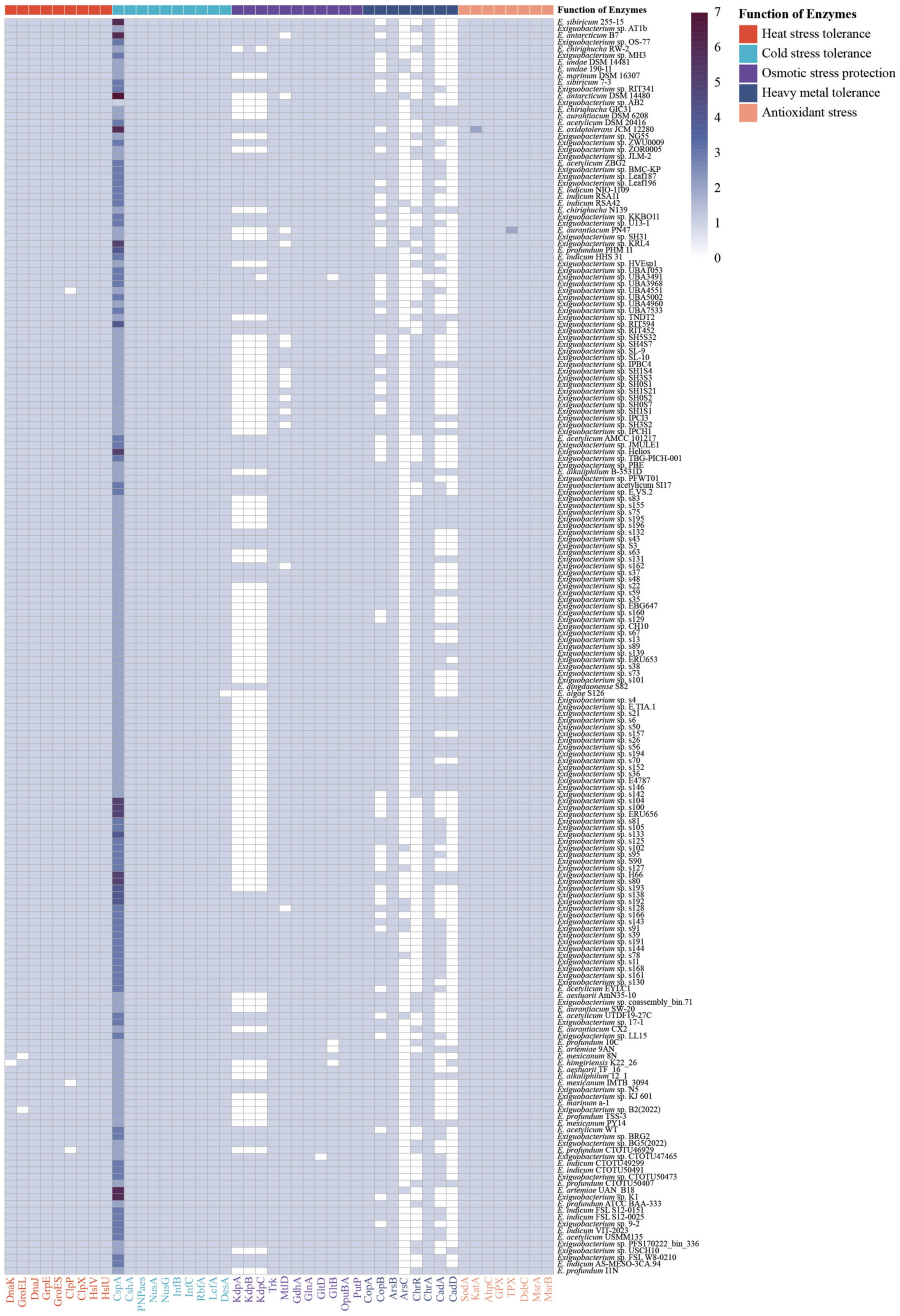
Distribution of enzymes with functions related to environmental adaptation in 187 strains of *Exiguobacterium*. Enzymes associated with heat stress tolerance, DnaK, GroEL, DnaJ, GrpE, GroES, ClpP, ClpX, HslV, and HslU; cold stress tolerance, CspA, CshA, PNPaes, NusA, NusG, InfB, InfC, RbfA, LcfA, and DesA; potassium ion transporters, KdpA, KdpB, KdpC, and Trk; compatible solute synthases, MtlD, GdhA, GlnA, GltD, GltB, OpuBA, and PutP; heavy metal resistance enzymes, CopA, CopB, ArsB, ArsC, ChrR, ChrA, CadA, and CadD; antioxidant stress kinases, SodA, KatA, AhpC, GPX, TPX, DsbC, MsrA, and MsrB.

*Heat stress:* Bacteria regulate the production and expression of heat-shock proteins to cope with the deleterious effects of heat stress (Singh et al., 2024). Heat-shock proteins function in two main ways. One is to use molecular chaperones (DnaK and GroEL), together with their co-chaperones (DnaJ-GrpE and GroES), to promote the proper folding of substrate polypeptides (Hayer-Hartl et al., 2016; Roncarati and Scarlato, 2017; Xiao et al., 2024). The *dnaK, dnaJ, grpE, groEL*, and *groES* genes were found in 186, 187, 187, 185, and 187 genomes of *Exiguobacterium* strains, respectively. Another way is to use proteases to remove damaged polypeptides from stressed cells (Roncarati and Scarlato, 2017). The ClpXP and HslUV proteases consist of the substrate recognition subunits (ClpX and HslU) and the catalytic subunit peptidases (ClpP and HslV; Joshi et al., 2004; Ogden et al., 2019). The *clpP, clpX, hslV*, and *hslU* genes were detected in 184, 187, 187, and 187 *Exiguobacterium* strains, respectively (Figure 3; Supplementary Table S1).

*Cold stress:* Several adaptive mechanisms are activated in bacteria at low temperatures to maintain transcription, translation and membrane fluidity (D'Amico et al., 2006). The *cspA* gene encoding cold-shock protein (CspA), which promotes translation and transcription by preventing secondary RNA structures formed at low temperatures (Jiang et al., 1997), was found in the genomes of 187 *Exiguobacterium* strains. The *cshA* gene, which promotes the unwinding of folded RNA at low temperatures to ensure continued translation, was identified in 187 strains (Lehnik-Habrink et al., 2010). The *pnp* gene encoding polynucleotide phosphorylase (PNPaes), which is essential for cell growth at low temperatures, was also found in all strains (Awano et al., 2008). Furthermore, several genes encoding transcription (*nusA* and *nusG*) and translation (*infB, infC*, and *rbfA*) factors that might contribute to RNA and protein synthesis in the cold (Dall'Agnol et al., 2014; Spaniol et al., 2013), and they were detected in all 187 *Exiguobacterium* genomes. Long-chain-fatty-acid-CoA ligase (LcfA) and desaturase (DesA) maintain cell membrane fluidity at low temperatures by degrading long-chain fatty acids and synthesizing unsaturated fatty acids, respectively (Adams et al., 2021; Dall'Agnol et al., 2014). The *lcfA* and *desA* genes were found in the genomes of 187 and 186 *Exiguobacterium*, respectively (Figure 3; Supplementary Table S1).

*Osmotic stress:* Microorganisms generally use two main strategies to maintain intracellular and extracellular osmotic balance (Gunde-Cimerman et al., 2018). One strategy is to accumulate potassium in the cytoplasm, which relies on the K^+^ uptake transporter proteins, including the Kdp and Trk system (Corratgé-Faillie et al., 2010; Epstein and Stock, 2016). The genes related to the Trk system were detected in all 187 strains of *Exiguobacterium*, while *kdpA, kdpB*, and *kdpC* genes were found in 91, 92, and 90 *Exiguobacterium* strains, respectively (Figure 3; Supplementary Table S1). Another strategy relies on the biosynthesis and accumulation of compatible solutes, such as mannitol, betaine, and glutamate (Zhao et al., 2023). The mannitol (*mtlD*) and glutamate (*gdhA, gltBD*, and *glnA*) biosynthetic genes were detected in the 173 and 187 strains of *Exiguobacterium*, respectively. In addition, genes encoding glycine betaine (*opuBA*) and proline (*putP*) transporters were found in all *Exiguobacterium* strains (Remonsellez et al., 2018).

*Oxidative stress:* Bacteria have two primary mechanisms for reactive oxygen species (ROS) detoxification (Zhao et al., 2024). One is to scavenge the ROS by enzymes, including superoxide dismutase (SOD), catalase (CAT), and peroxidase (Johnson and Hug, 2019). The *sodA* gene, which catalyzes the conversion of superoxide anions (${\text{O}}_{2}^{-}$) to O_2_ and hydrogen peroxide (H_2_O_2_; Zhang et al., 2019), was found in the genomes of 187 *Exiguobacterium* strains. The *katA* gene, which is responsible for the conversion of H_2_O_2_ (Imlay, 2013), was found in all 187 strains. The genes encoding alkylhydroperoxidase (AhpC), glutathione peroxidase (GPX) and thioredoxin peroxidase (TPX), which catalyze the reduction of many oxidants (Panday et al., 2020; Vasková et al., 2023), were also present in all strains of *Exiguobacterium*. Another is the use of disulfide isomerase (DsbC) and methionine sulfoxide reductase (Msr) to repair cysteine and methionine residues oxidized by ROS, respectively (Darby et al., 1998; Grimaud et al., 2001). The genes *dsbC, msrA*, and *msrB* were identified in all strains (Figure 3; Supplementary Table S1).

*Heavy metal tolerance:* To survive in heavy metal-polluted environments, bacteria have evolved resistance mechanisms to metabolize heavy metals (Pal et al., 2022). The CopAB proteins are responsible for resistance to copper. The *copA* gene encodes CopA, which is an effective copper pump at low and high copper concentrations, was found in all 187 strains of *Exiguobacterium*, while the *copB* gene encodes CopB, a low-affinity copper exporting ATPase that is only relevant at high copper concentrations, was found in 133 strains (Völlmecke et al., 2012). The *ars* operon is responsible for bacterial resistance to arsenic. Arsenate reductase (ArsC) catalyzes the reduction As(V) (arsenate) to As(III) (arsenite), and then As(III) is directly pumped out of the cell by ArsB (Hao et al., 2021). The *arsC and arsB* genes were found in 15 and 187 strains, respectively (Figure 3; Supplementary Table S1). The *chr* operon promotes bacterial resistance to chromium. Among them, the chromate reductase ChrR catalyzes the conversion of Cr(VI) to Cr(III), while the ChrA protein pumps out intracellular chromate (Baldiris et al., 2018; Díaz-Pérez et al., 2007). The *chrR* gene was found in 102 strains of *Exiguobacterium*, while the *chrA* gene was found in 186 strains and was only absent in the genomes of *Exiguobacterium* sp. UBA3491. In addition, cadmium efflux transporter genes *cadA* and *cadD* were detected in 82 and 41 strains, respectively (Korir et al., 2022; Liu et al., 2021).

*Antibiotic resistance:* Bacteria usually harbor many antibiotic resistance genes (ARGs) to cope with antibiotic stress in the environment (Zhuang et al., 2021). A total of 2,596 antibiotic resistance genes were identified, with the number of ARGs per strain of *Exiguobacterium* ranging from 10 to 20 (Supplementary Table S5). The *gyrB* gene related to fluoroquinolone resistance was detected in 187 *Exiguobacterium* strains, while *rpoB* gene associated with rifampicin resistance was found in 185 strains. *mdtG*, an MFS efflux pump gene involved in the resistance to phosphonic acid antibiotic, was identified in all 186 strains except for *E. alkaliphilum* 12_1. *tet*(*35*), an ABC efflux pump gene cluster involved in the resistance to tetracyclin antibiotics, was found in the genomes of 176 *Exiguobacterium* strains. The highest numbers of antibiotics associated with resistance mechanisms were fluoroquinolones, rifamycin, and tetracyclines, with 639, 324, and 298 resistance proteins, respectively, while carbapenem, fusidane, and mupirocin-like had a low number of resistance genes.

### Genomic islands (GIs) in *Exiguobacterium*

Microorganisms can evolve and adapt through horizontal transfer of MGEs, including phages, genomic islands (GIs), and associated elements (Sobecky and Hazen, 2009). A total of 1,722 GIs were predicted in the 187 *Exiguobacterium* strains. The number of GIs per strain ranged from 2 to 22 (Supplementary Table S1). *Exiguobacterium* sp. E4787 and *E. mexicanum* PY14 contained the highest number of GIs (22), while *Exiguobacterium* sp. UBA7533, *Exiguobacterium* sp. PFWT01, *E. profundum* 10C, and *E. marinum* a-1 contained the fewest (2). There are many vital functional protein genes in these GIs, including glycosyltransferase (133 strains), kinase (131 strains), sugar transferase (100 strains), heavy metal translocating P-type ATPase (88 strains), response regulator transcription factor (78 strains), type II toxin-antitoxin system (67 strains), nucleotide sugar dehydrogenase (62 strains), MFS transporter (55 strains), cadmium resistance transporter (43 strains), metallopeptidase (40 strains), copper resistance protein (39 strains), CDF family zinc transporter CzcD (32 strains), RNA polymerase (31 strains), mercury resistance protein (27 strains), and iron ABC transporter (25 strains; Supplementary Table S6). These genes are involved in the physiology at the transcriptional and translational levels, and confer heavy metal resistance to the strains.

### Overview and taxonomy of phages in *Exiguobacterium*

To determine the prevalence of phage in *Exiguobacterium* genomes, we screened the genomes of 187 *Exiguobacterium* strains using a combination of several different methods. In total, 58 putative phage contigs were identified in 26.2% (49/187 strains) of the *Exiguobacterium* genomes (Supplementary Table S7). In addition, 41 uncultured viral genomes (UViGs) associated with *Exiguobacterium* were collected in the IMG/VR v.4 dataset (Camargo et al., 2023) and verified to infect the genus *Exiguobacterium* by iPHoP. All 99 viral sequences were clustered with 95% ANI and 80% coverage, resulting in 65 viral operational taxonomic units (vOTUs, Supplementary Table S7). The genome size of vOTUs ranged from 5,258 to 121,308 bp, and 96.9% of them were between 5 and 50 kb (Supplementary Table S7). Of these 65 vOTUs, 2 (3.1%), 18 (27.7%), 22 (33.8%), 21 (32.3%), and 2 (3.1%) were complete, high-quality, medium-quality, low-quality, and quality not-determined genomes, respectively (Supplementary Table S8).

**Figure 4:**
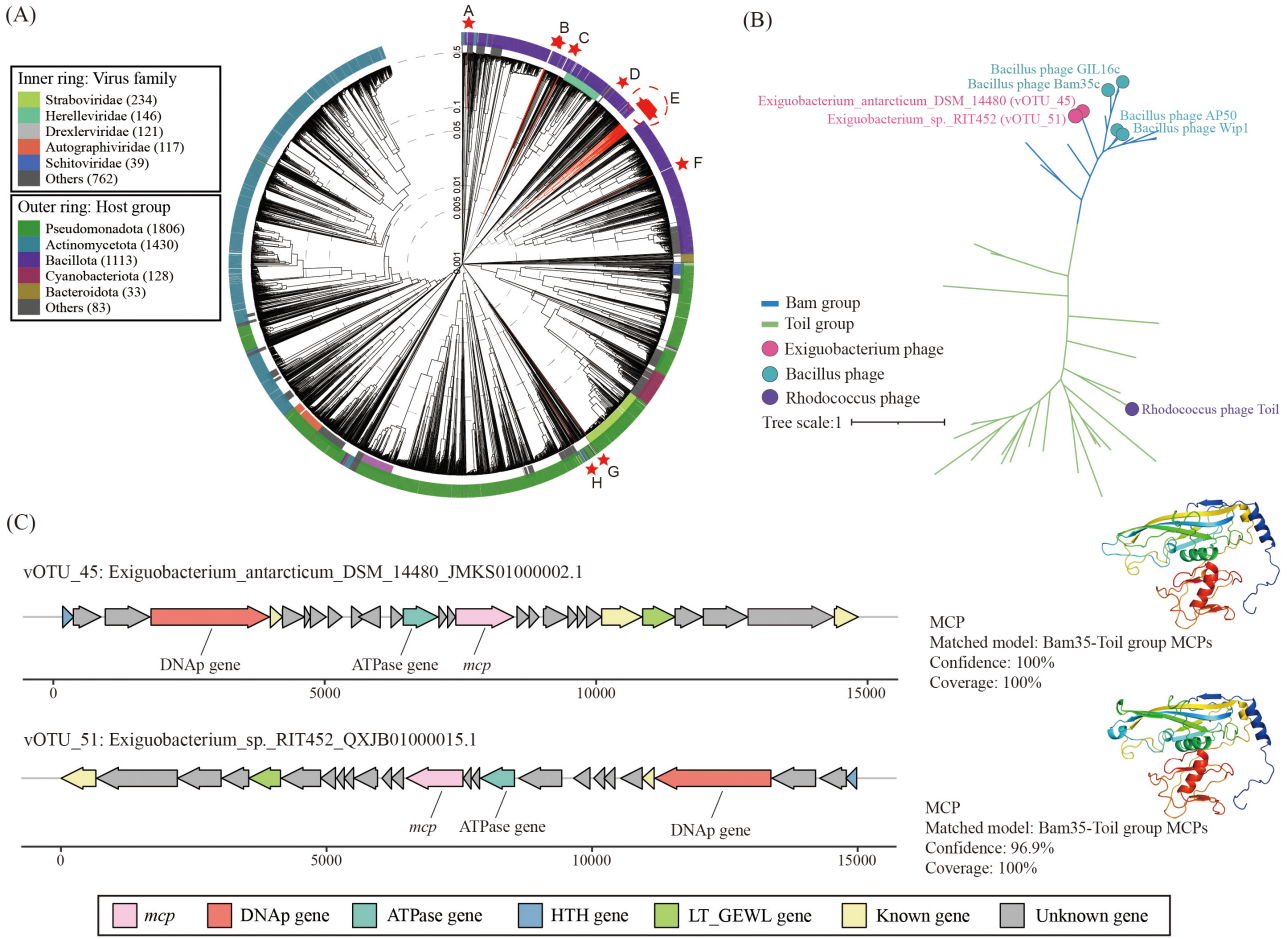
**(A)** The genome-wide proteomic tree of *Exiguobacterium* phages and other related known dsDNA phages constructed using ViPTree. The inner and outer colored rings indicate viral family and host groups, respectively. Phages identified in this study are marked with red stars, with corresponding branches highlighted in red. The eight major clades containing *Exiguobacterium* phages are denoted A–H. **(B)** Maximum likelihood tree of the MCP proteins. Branch color indicates different double jelly-roll major capsid proteins (DJR MCPs) groups. **(C)** Genome architecture of putative phages encoding DJR MCPs and the protein structure of their DJR MCPs. Gene abbreviations: MCP, major capsid protein; DNAp, DNA polymerase; HTH, helix-turn-helix domain-containing protein; LT_GEWL, lytic transglycosylase domain-containing protein.

Taxonomic assignment showed that 65 vOTUs were classified in two different viral realms, that is, *Duplodnaviria* (63 vOTUs, viruses with double-stranded DNA genomes) and *Varidnaviria* (2 vOTUs, a portmanteau of various DNA viruses). All classified vOTUs were resolved at the class level (Supplementary Table S7). The viral proteome tree constructed based on genome-wide sequence similarity showed 65 vOTUs distributed in eight clades, many of which are far from known prokaryotic double-stranded DNA (dsDNA) virus families (Figure 4A). The largest group (clade E) included 46 vOTUs, followed by clades B and C comprising seven and four vOTUs, respectively. Only clade D, G, and H contained one vOTU, which was unrelated to each other. In addition, these vOTUs were further classified at the family level using PhaGCN2. As a result, only 36.9% (24) vOTUs were classified at the established family level, including *Azeredovirinae* (10, 15.4%), *Peduoviridae* (6, 9.2%), *Joanripponvirinae* (4, 6.2%), *Tectiviridae* (2, 3.1%), *Casjensviridae* (1, 1.5%), and *Aliceevansviridae* (1, 1.5%; Supplementary Table S7), suggesting that these *Exiguobacterium* phages may have significant phylogenetic novelty.

Apart from diverse tailed phages belonging to *Duplodnaviria*, double jelly-roll major capsid protein (DJR MCP) encoding sequences were identified in two vOTUs, which represent non-tailed dsDNA phages of the *Varidnaviria*. The phylogenetic tree indicated that these two DJR MCPs clustered with the Bam35-Toil group MCPs (Figure 4B). This group was divided into Bam35 and Toil subgroups, which were mainly related to phages infecting *Terrabacteria* (*Actinobacteria* and *Firmicutes*; Yutin et al., 2018). Protein structural homology searches also showed that the MCPs of vOTU_45 (100% confidence and coverage) and vOTU_51 (96.9% confidence and 100% coverage) were most similar to the Bam35-Toil group MCPs (Figure 4C). In addition, genes encoding MCP, ATPase, and DNAp were found in both phage genomes, which is consistent with most reported Bam35-Toil group phage (Yutin et al., 2018).

### Putative auxiliary metabolic genes (AMGs) in *Exiguobacterium* phages

**Figure 5:**
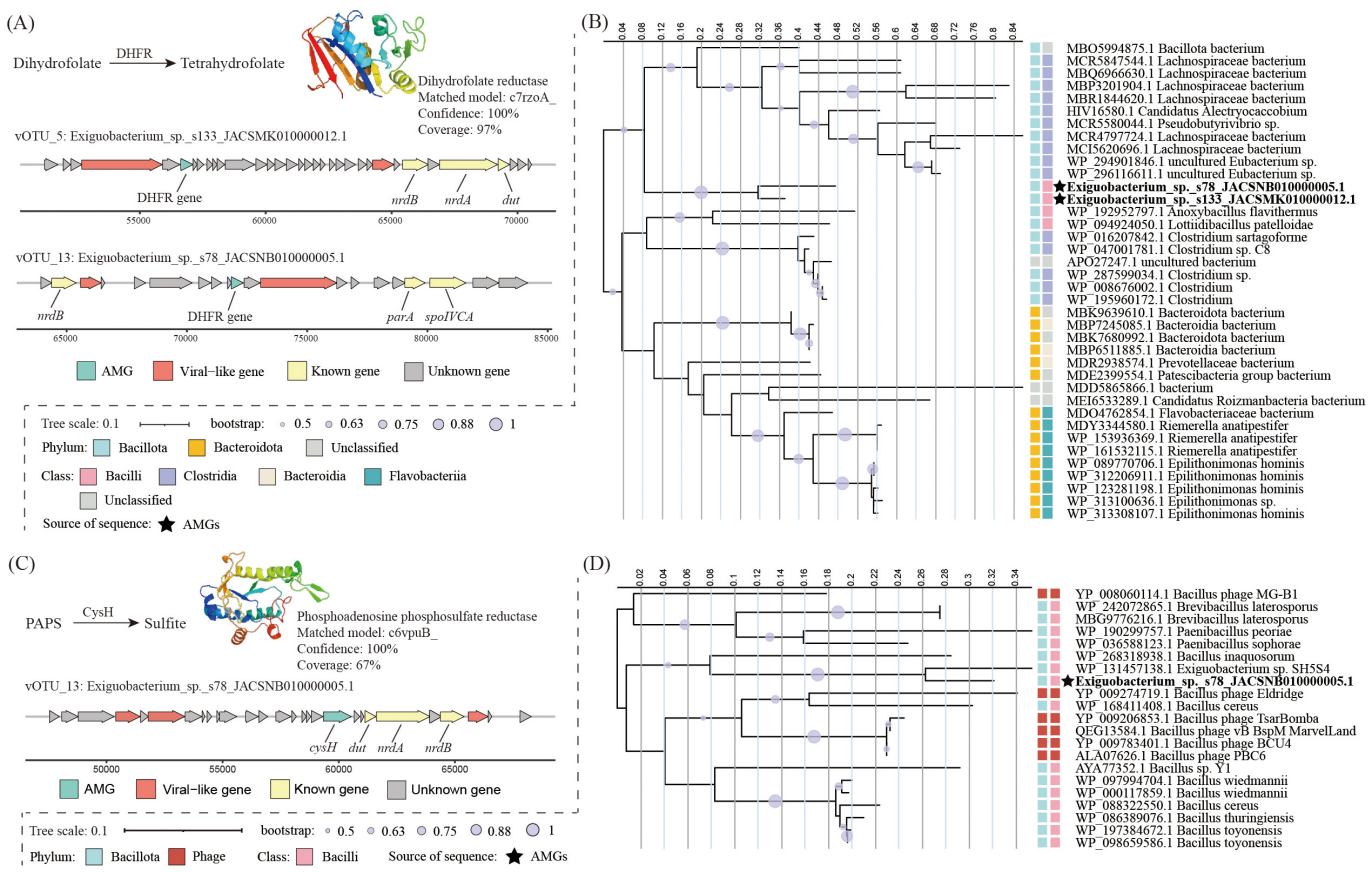
**(A)** Metabolic pathways with DHFR genes, the reference protein models for the DHFR, and genome architecture of vOTU encoding DHFR genes. **(B)** The phylogenetic tree of DHFR. **(C)** Metabolic pathways with *cysH* genes, the reference protein models for the CysH, and genome architecture of vOTU encoding *cysH* genes. **(D)** The phylogenetic tree of CysH.

Using the DRAM-v and VIBRANT pipelines, three putative AMGs were identified in two *Exiguobacterium* phage genomes (Figure 5). These AMGs were involved in two functional categories, that is, metabolism of cofactors and vitamins and energy metabolism. The predicted AMGs were all surrounded by phage genes (DRAM-v auxiliary scores < 4), suggesting that they were truly of viral origin. Putative AMGs encoding dihydrofolate reductase (DHFR) involved in cofactor and vitamin metabolism were identified in vOTU_5 and vOTU_13. Protein structure modeling supported (100% confidence) these two proteins as DHFR (Figure 5A). Phylogenetic analysis showed that the DHFR encoded by the two phages closely clustered with the reference sequences of the Clostridia (Figure 5B). In addition, the AMG was assigned to phosphoadenosine phosphosulfate reductase (CysH) involved in sulfur metabolism, which was detected in the vOTU *Exiguobacterium*_sp._s78_JACSNB010000005.1. Protein structure modeling verified it with 100% confidence as CysH (Figure 5C), a member of the assimilatory sulfate reduction pathway that catalyzes the reduction of 3′-phosphoadenosine 5′-phosphosulfate (PAPS) to sulfite. Phylogenetic analysis revealed that *Exiguobacterium* phage-encoded CysH most closely with several reference sequences from Bacilli and phage (Figure 5D).

## Discussion

The genus *Exiguobacterium* is known for its widespread distribution in diverse habitats on Earth, including extreme environments (Kasana and Pandey, 2018). This wide distribution indicates that *Exiguobacterium* strains have evolved multiple adaptive strategies to survive in these challenging environments. In this study, we systematically and comprehensively investigated the environmental adaptation mechanisms of *Exiguobacterium* members using the comparative genomic method. Our results show that *Exiguobacterium* strains have a remarkable ability to utilize various nutrients, especially carbon and nitrogen sources, which is consistent with previous findings but further supported here by a larger and systematically analyzed genome dataset (Shen et al., 2024; Vishnivetskaya et al., 2009; Zhang et al., 2021). Specifically, all 18 genomes encoded intact glycolysis, TCA cycle, and pentose phosphate pathways, indicating a conserved capacity for utilizing monomeric carbohydrates among the analyzed strains. In addition, these genomes contained key genes for the PTS, further supporting their potential for efficient carbohydrate uptake and utilization in complex environments. Consistent with this, multiple CAZymes were identified in all 187 strains, suggesting the capacity to degrade a range of complex polysaccharides, including cellulose, hemicellulose, starch, and chitin, which are widely distributed in natural environments (Klemm et al., 2005; Pellis et al., 2022; Scheller and Ulvskov, 2010; Tanackovic et al., 2016).

In contrast to the conserved carbon metabolic pathways, none of the 18 analyzed genomes encoded complete pathways for inorganic nitrogen assimilation or inorganic sulfate reduction. While previous studies have emphasized the broad metabolic capabilities of *Exiguobacterium*, our results further suggest a potential limitation in inorganic nitrogen and sulfur utilization suggesting that the *Exiguobacterium* strains are unlikely to rely on inorganic nitrate or sulfate as sole nitrogen or sulfur sources and may instead depend on organic forms such as amino acids. Consistent with this inference, numerous secreted peptidases were identified across the analyzed genomes, indicating a potential capacity to metabolize and utilize organic nitrogen sources from the environment. Among them, cysteine peptidase C26, serine peptidases S33 and S09, and metallo peptidase M38 were the most abundant peptidases. The peptidase C26 reportedly enhances the ability of bacteria to utilize glutamine and glutathione (Hofreuter et al., 2008), and M38 catalyzes the hydrolytic cleavage of isoasparaginyl dipeptide (Martí-Arbona et al., 2005). S33 peptidase catalyzes the proline removal from the N-terminus of peptides or proteins (Dong et al., 2022), while S09 peptidase cleaves the peptide at the C-terminal side of the proline residue (García-Horsman et al., 2007).

In addition to genes required for energy metabolism, the genomes of *Exiguobacterium* strains contained genes related to various stress resistance systems. For example, nearly all *Exiguobacterium* strains harbored genes encoding heat-shock proteins, including molecular chaperones (DnaK–DnaJ–GrpE and GroEL–GroES) and proteases (ClpXP and HslUV), which are involved in protein folding and the removal of damaged polypeptides under heat stress, consistent with previous reports of strong stress tolerance in this genus (Zhang et al., 2021). Notably, cold adaptation mechanisms in this genus are equally diverse and evolutionarily conserved. Previous studies have shown that *E. antarcticum* B7 can respond to cold stress via expression of cold shock protein genes, regulation of transcription and translation, and chemical modification of the cell membrane (Dall'Agnol et al., 2014). Our findings extend these observations, revealing that such cold adaptation strategies are not limited to psychrotrophic species but are ubiquitously present in the entire genus of *Exiguobacterium*. In addition, *Exiguobacterium* employs a dual strategy combining ion homeostasis and compatible solute accumulation to counteract osmotic stress. Specifically, the Trk potassium uptake system, universally conserved in all 187 strains, enables rapid accumulation of cytoplasmic K^+^ to balance extracellular hyperosmolarity. All of them also harbored genes related to the biosynthesis (*mtlD, gdhA/gltBD/glnA*) and translocation (*opuBA, putP*) of compatible solutes, such as mannitol, glutamate, glycine betaine, and proline (Remonsellez et al., 2018). These compatible solutes not only maintain cellular osmotic homeostasis, but also act as stabilizers of proteins and cellular components, preventing denaturing effects of high ionic strengths (Kempf and Bremer, 1998).

Heavy metals are among the most prevalent chemical pollutants in natural environments and exert strong toxicity on microbial communities (Feng et al., 2018). To overcome this pressure, some bacteria have evolved defense systems that either expel toxic compounds or convert them into less harmful forms (Biswas et al., 2021). Genomic analysis of *Exiguobacterium* strains revealed their ability to mitigate heavy metal toxicity through these mechanisms, with resistance genes primarily associated with transporters including *copA, arsB*, and *chrA*, which were conserved in more than 99% of the analyzed strains. This transporter-centric profile suggests that *Exiguobacterium* may adapts to heavy metal stress primarily through rapid ion expulsion rather than enzymatic detoxification. Notably, several resistance genes, including *copB, arsC, chrR, cadA*, and *cadD*, exhibited uneven distributions among the analyzed genomes, which were isolated from diverse environments rather than exclusively from metal-contaminated habitats. This pattern suggests that the presence of these genes does not necessarily reflect adaptation to high concentrations of heavy metals, but may instead be associated with exposure to low levels of metals that are ubiquitous in natural environments (Gadd, 2010). Furthermore, this distribution implies that these genes may have been acquired via HGT (Patra et al., 2025).

In addition, ARGs were also identified in *Exiguobacterium*, primarily associated with antibiotic target alteration and multidrug efflux, both of which contribute to survival under antibiotic stress. Specifically, target modification genes such as fluoroquinolone-resistant *gyrB* and rifampicin-resistant *rpoB* were widely distributed, reducing antibiotic binding affinity by altering the structure of DNA gyrase and RNA polymerase (Campbell et al., 2001; Caspar et al., 2017). Nearly all *Exiguobacterium* strains also harbored the MFS and ABC multidrug efflux pumps, which enable the export of structurally diverse antibiotics, including tetracyclines, fluoroquinolones, macrolides, β-lactams, and rifamycins, thereby reducing intracellular drug concentrations (Du et al., 2018; Li et al., 2015). Furthermore, GIs analysis indicates that numerous metal resistance and multidrug efflux systems are embedded within these horizontally acquired regions, highlighting the critical role of HGT in shaping the adaptive repertoire of *Exiguobacterium*. Remarkably, 18 terpene-related smBGCs showed no similarity to any known clusters in public databases, suggesting that they may represent novel terpene biosynthetic pathways. These findings indicate that *Exiguobacterium* harbors previously uncharacterized metabolic potential, which may contribute to its ecological adaptability across diverse environments. In summary, the genes related to carbon and nitrogen source utilization, heat and cold stress, antioxidative stress, osmotic protection, antibiotic and heavy metal resistance enhance the survival ability of the strains of *Exiguobacterium* to survive in extreme environments, as well as promote their potential for widespread distribution.

Beyond core genome–encoded adaptive traits and horizontally acquired GIs, bacteriophages represent another important driver of genome diversification and ecological adaptation in bacteria. In this study, phage sequences were detected in approximately one quarter of the analyzed *Exiguobacterium* genomes, indicating that phage integration is a recurrent but non-universal feature within this genus. This relatively low prevalence may be associated with the presence of diverse defense mechanisms encoded by *Exiguobacterium*, including CRISPR–Cas systems, which are known to restrict the integration and maintenance of foreign genetic elements (Cañiza and Dy, 2025; Shen et al., 2024). For example, a prokaryotic Argonaute protein (EsAgo) has been reported in *Exiguobacterium* sp. AB2, which is encoded in close proximity to other defense systems within genomic “defense islands,” underscoring the robust antiviral arsenals of this genus (Cañiza and Dy, 2025).

Additionally, 63.1% of vOTUs could not be assigned to known viral families, revealing substantial genomic novelty and highlighting the underexplored diversity of *Exiguobacterium*-associated phages. Notably, AMGs encoding DHFR and CysH were identified in two phage genomes. DHFR catalyzes the reduction of dihydrofolate to tetrahydrofolate, a key cofactor in one-carbon metabolism that supports nucleotide biosynthesis and DNA replication, thereby potentially facilitating efficient viral genome synthesis during infection (Lo et al., 2024). CysH catalyzes a key step in sulfate assimilation, providing reduced sulfur for methionine and cysteine biosynthesis in microorganisms (Kredich, 2008). Interestingly, although some *Exiguobacterium* strains encode the *cysH* gene, the specific host strain of this phage lacks it. One possible explanation is that the host may rely on organic sulfur sources available in its environment and has consequently lost part of the assimilatory sulfate reduction pathway during genome streamlining (Giovannoni et al., 2014). In such cases, phage-encoded *cysH* may transiently complement this metabolic deficiency during infection, ensuring sufficient sulfur supply for nucleotide and protein synthesis required for viral replication (Kieft et al., 2021). This phenomenon is consistent with the concept of AMGs, whereby phages carry host-derived metabolic genes to modulate host physiology and optimize intracellular conditions for viral replication (Breitbart, 2012). Collectively, the presence of these metabolically relevant genes suggests that certain *Exiguobacterium*-associated phages may reprogram host metabolism to optimize intracellular conditions for viral replication, while also influencing host biosynthetic capacity.

## Conclusions

Comparative genomic analysis of 187 *Exiguobacterium* strains revealed extensive genetic features that enable their adaptation to diverse environments and broad global distribution. Specifically, nearly all strains harbor the capacity to utilize ubiquitous carbohydrates and proteinaceous as carbon and nitrogen sources, reflecting strong metabolic versatility. Furthermore, to resist and adapt to external environmental stresses, the genomes of all strains accumulated numerous functional genes associated with heat and cold tolerance, antioxidant stress, osmotic pressure protection, heavy metal, and antibiotic resistance. In addition to these core adaptive traits, GIs contribute to ecological flexibility by introducing horizontally acquired genes involved in resistance, regulation, and metabolic functions, while phages further enhance genomic diversity and functional potential through integration events and the occasional carriage of AMGs. Overall, these combined genomic features provide a genetic foundation that supports environmental resilience and promotes the widespread distribution of *Exiguobacterium* across diverse and often extreme habitats.

## Data Availability

The original contributions presented in the study are included in the article/Supplementary material, further inquiries can be directed to the corresponding author.
