# Supplementary material for: Comprehensive genomic analyses revealed the adaptation strategies of Exiguobacterium and its phage genomic diversity
Source: Front Microbiol. 2026 May 21;17:1841508. doi: 10.3389/fmicb.2026.1841508 (PMC13233415; doi:10.3389/fmicb.2026.1841508)
Supplement: Supplementary file 1 [file Data_Sheet_1.pdf]

Supporting information to:

Comprehensive genomic analyses revealed the adaptation strategies of *Exiguobacterium* and its phage genomic diversity

Yiping Tian[1*], Leyi Zou[2], Yu Ji[3]

[1] Department of Geography, Xinzhou Normal University, Xinzhou, 034000, China
[2] First Clinical Medical College, Gansu University of Chinese Medicine, Lanzhou, 730000, China
[3] Lab for Microbial Resources, School of Ecology and Environment, Inner Mongolia University, Hohhot, 010021, China.

This PDF file includes:

Figures S1 to S3

Supplementary figures

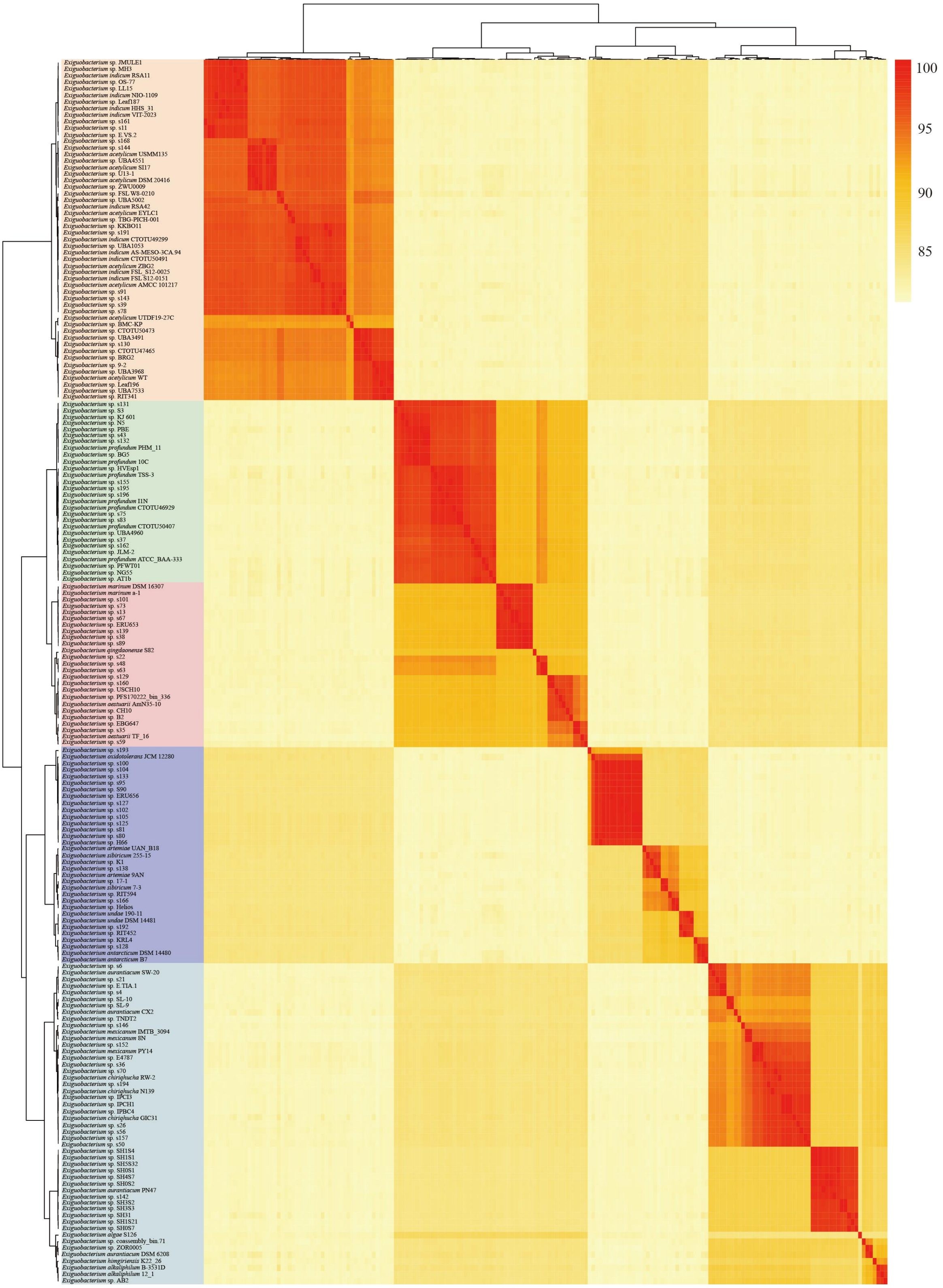

Figure S1 Pairwise average nucleotide identity comparison between the genomes of 187 strains of *Exiguobacterium*. The ANI values were used to construct a dendrogram and a heatmap with the average linkage method and Euclidean distance used for clustering and correlation analyses, respectively.

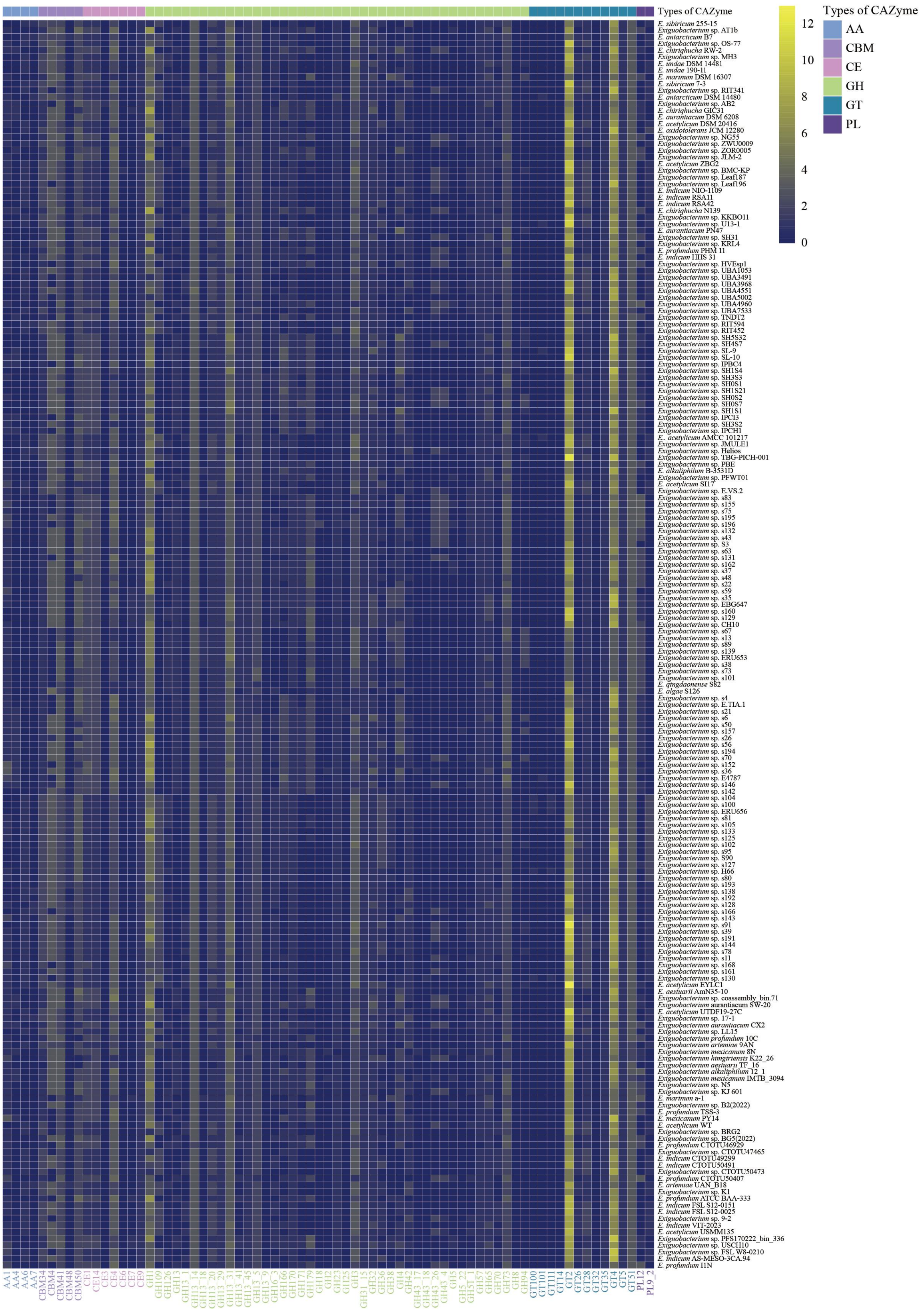

Figure S2 Distribution of the carbohydrate-active enzymes (CAZymes) in the genome of *Exiguobacterium*. AA, auxiliary activities; CBM, carbohydrate-binding module; CE, carbohydrate esterase; GH, glycoside hydrolase; GT, glycosyltransferase; PL, polysaccharide lyase.

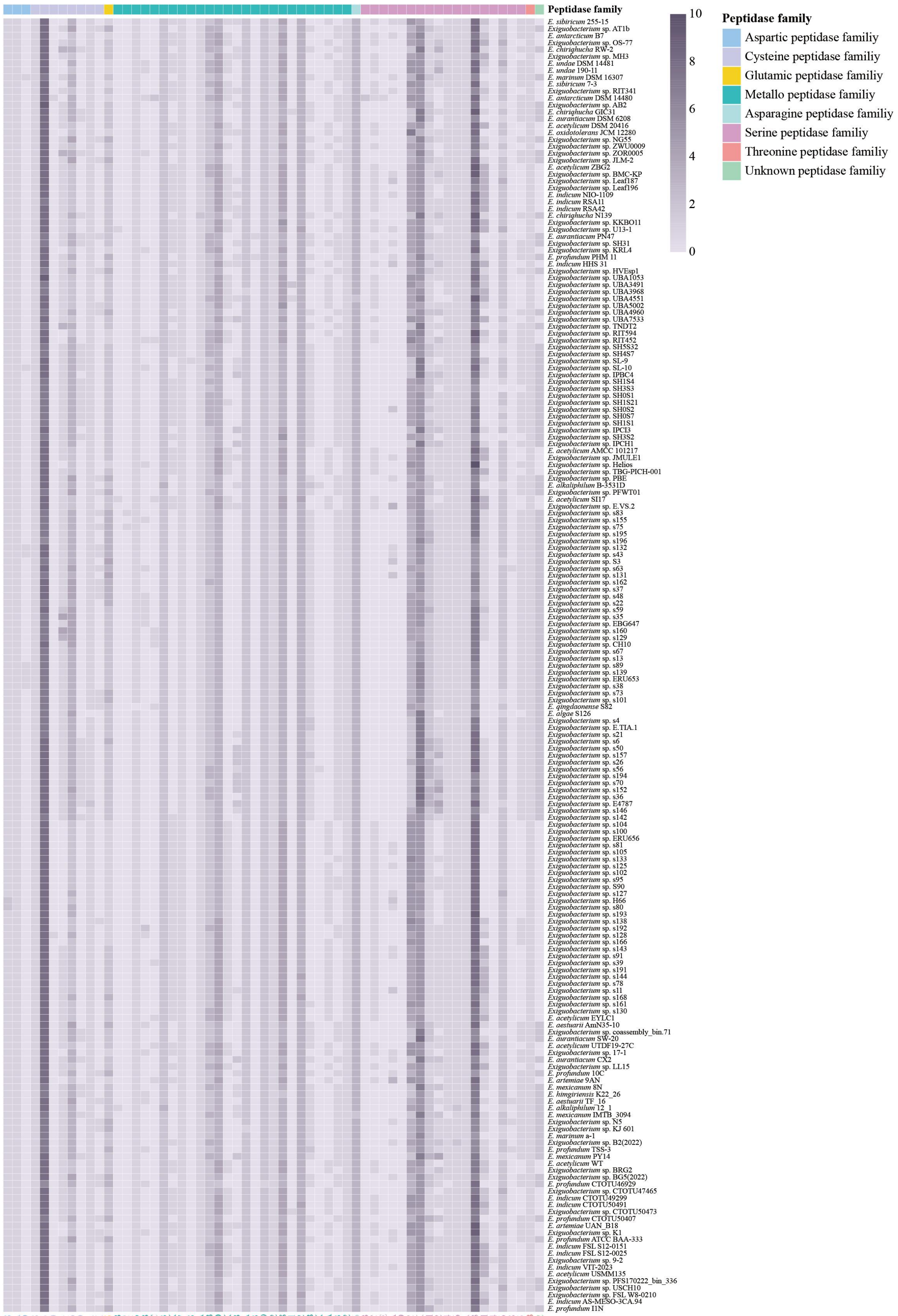

Figure S3 Distribution of the peptidases in the genome of *Exiguobacterium*.
